# Approach to Cefepime-Induced Neurotoxicity in the Setting of Chronic Kidney Disease: A Case Report and Review of Literature

**DOI:** 10.7759/cureus.26005

**Published:** 2022-06-16

**Authors:** Ariel Ruiz de Villa, Kipson Charles, Raghav Bassi, Sanjae Spencer, Yvette Bazikian

**Affiliations:** 1 Internal Medicine, University of Central Florida College of Medicine, Graduate Medical Education/Hospital Corporation of America (HCA) Healthcare North Florida Regional Medical Center, Gainesville, USA

**Keywords:** hemodialysis, chronic kidney disease, cefepime, comatose, neurotoxicity

## Abstract

Cefepime-induced neurotoxicity is well-known, but an under-recognized event that can present with a myriad of neurological findings ranging from myoclonus to seizures to comatose state. It is more prevalent in patients with impaired renal clearance as it is mainly cleared by the kidneys. We present a case of a 52-year-old female who was managed in the intensive care unit with severe encephalopathy following empiric antibiotic therapy with cefepime. Although we encountered some unforeseen difficulties executing our initial plan of renal replacement therapy, our patient was successfully treated with fluids and intravenous diuresis with furosemide and was ultimately discharged home with full recovery.

## Introduction

Cefepime is a β-lactam antibiotic classified as a fourth-generation cephalosporin. It is highly effective against both Gram-positive and Gram-negative bacteria, is widely administered parenterally, and is often one of the first choices by clinicians due to its wide spectrum of coverage. A well-known but rarely recognized and underdiagnosed adverse effect of cefepime is neurotoxicity, particularly seen in patients with renal impairment. These patients are at increased risk because hepatic metabolism of cefepime is minimal with 85% of the drug excreted in the urine unchanged [1]. Herein, we present a case of a patient with acute kidney injury (AKI) with a baseline chronic kidney disease stage 3 (CKD) who was empirically treated with cefepime for community-acquired pneumonia and consequently developed significant neurologic toxicity, requiring management in the critical care setting. A literature review was conducted as we discuss our approach to care and recommendations for the management of patients with similar presentations.

## Case presentation

The critical care service was consulted to evaluate a 52-year-old Caucasian female who had been admitted to the hospital two days prior with a chief complaint of acute hemoptysis and dyspnea. She was later diagnosed with community-acquired pneumonia and subsequently treated with IV cefepime. The patient’s condition deteriorated and she was subsequently found to be unresponsive with new myoclonic jerks of the upper and lower extremities. Her medical history was significant for granulomatosis with polyangiitis, on immunosuppressive therapy with mycophenolic acid, tracheal stenosis requiring tracheostomy, and 5 liters (L) supplemental oxygen use, type 1 diabetes mellitus, chronic kidney disease stage 3b, peripheral neuropathy, and hypothyroidism. She was retired and denied any recreational drug use or alcohol consumption at the time of admission.

Upon evaluation, vitals were as follows: blood pressure was 122/71 mmHg, respiratory rate was 20 rpm, pulse rate was 74 bpm, pulse oximetry was 90%, and temperature was 95°F. The patient was unresponsive, intermittently and non-purposely opening her eyes, exhibiting rhythmic myoclonic jerks more prominent in her upper and lower extremities. She was unable to follow commands or communicate, with a Glasgow Coma Scale (GCS) of 5 (1 eye, 1 verbal, 3 motor). A tracheostomy collar was in place with 15 L of oxygen delivered per minute. Breath sounds were coarse bilaterally with minimal elevation of the chest during inspiration. According to the bedside nurse report, the patient had been alert and oriented, able to endorse shortness of breath a few hours earlier. The patient’s current medications were reviewed for any offending agents that could be contributing to the patient’s mental status change (Table 1). Laboratory results from hours earlier were reviewed as well (Table 2). Urinalysis revealed glucosuria and proteinuria, which was otherwise unremarkable.

**Table 1:** Patient’s current medications at the time of initial assessment

Medication Name	Dose/Frequency
Levothyroxine	88 mcg daily
Budesonide	0.25 mg twice daily inhaled
Metoprolol succinate	25 mg daily
Amlodipine	10 mg daily
Atorvastatin	20 mg daily
Gabapentin	300 mg three times daily
Levosalbutamol	1.25 mg four timed daily inhaled
Regular insulin	As per sliding scale
Mycophenolate mofetil	1000 mg twice daily
Doxycycline	100 mg twice daily
Cefepime	2g twice daily

**Table 2:** Laboratory results available at the time of initial assessment MCV: mean corpuscular volume; BUN: blood urea nitrogen; GFR: glomerular filtration rate; AST: aspartate aminotransferase; ALT: alanine transaminase; TSH: thyroid-stimulating hormone

Tests	Results	References	Units
White blood count	15.4	4.0-10.5	10³ uL
Hemoglobin	8.3	13.7-17.5	g/dL
Hematocrit	26	40.1-51	%
Platelets	132	150-400	10³ uL
MCV	86.7	79.0-92.2	fL
Sodium	141	136-145	mmol/L
Potassium	5.1	3.5-5.1	mmol/L
Chloride	111	98-107	mmol/L
Carbon dioxide	25	21-32	meq/L
Glucose	200	74-106	mg/dL
BUN	73	7-18	mg/dL
Creatinine	2.29	0.6-1.30	mg/dL
GFR	24	0-120	mL/min
Albumin	2.3	3.4-5.0	g/dL
Calcium	8.8	8.5-10.1	mg/dL
Phosphorus	4.7	2.5-4.9	mg/dL
Total bilirubin	0.5	0.2-1.0	mg/dL
AST	17	15-37	units/L
ALT	18	13-56	units/L
Magnesium	2.7	1.8-2.4	mg/dL
Lactic acid	0.58	0.4-2.0	mmol/L
TSH	1.190	0.358-3.740	uIU/mL

**Figure 1:**
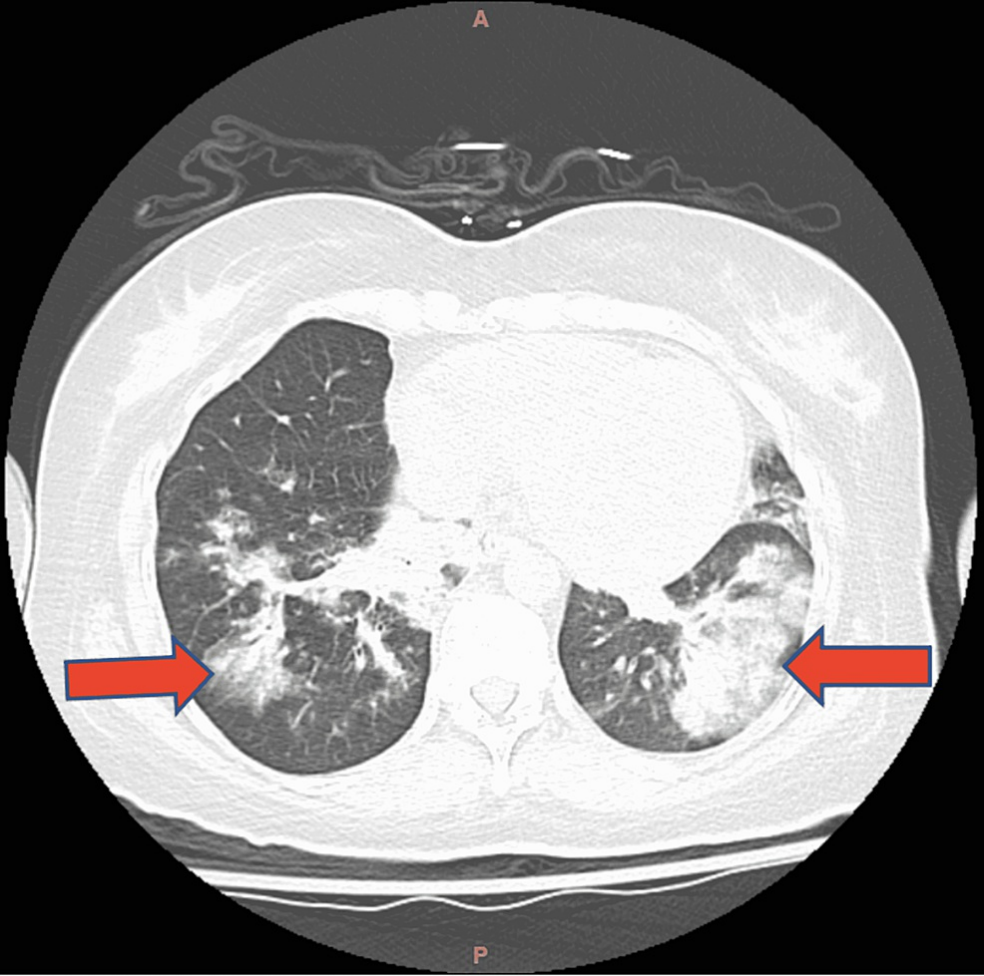
CT scan of patient's chest. Red arrows pointing at areas of disease consistent with bilateral pneumonia

Chest CT scan on admission revealed bilateral lower lobe infiltrates consistent with pneumonia (Figure 1). On admission, the patient was treated with intravenous (IV) cefepime and oral doxycycline for empiric respiratory tract infection. Bedside arterial blood gas was consistent with hypoxic respiratory failure (Table 3). 

**Table 3:** Arterial blood gas results with tracheostomy collar at 15 L O2/min

Test	Result	Reference	Units
pH	7.26	7.35-7.45	No units
pCO_2_	47	35-48	mmHg
pO_2_	69.9	83-108	mmHg
pO_2_/FiO_2_ ratio	199.7	Ratio	Ratio
HCO_3_	20.8	21-28	mmol/L
O2 saturation	90.7	94-98	%

For the past few years, the patient had been receiving treatment with the immune suppressant mycophenolic acid, for granulomatosis with polyangiitis. Since then, she had had recurrent mixed infections and hospitalizations. The patient’s husband was able to recall that in the past the patient had had adverse reactions to some antibiotics; however, he was not able to recall a specific drug. He alluded to the possibility of a penicillin derivative. This was not part of the list of allergies and medication intolerances on the patient’s chart.

Of note, the patient had been closely followed by a nephrologist for rapid decline in renal function. It was documented that the patient had a kidney biopsy showing diffuse and nodular diabetic nephropathy with severe arterio-arteriolar sclerosis and severe tubular compromise. After initial assessment and review, the patient was placed on a ventilator via tracheostomy access and transferred to the intensive care unit for further evaluation and management of encephalopathy, AKI superimposed on CKD, and acute on chronic hypoxic respiratory failure. Urinary output within 12 h was less than 0.2 cc/kg/h. The antibiotic regimen was changed to aztreonam, and mycophenolic acid was discontinued in the setting of infection. A bedside lumbar puncture was done given her rapid change in neurological function. Cerebrospinal fluid was negative for WBC, RBC, total proteins, herpes, and varicella DNA making infectious etiologies less likely. Given the lack of infectious or metabolic etiologies contributing to the patient’s neurological deterioration, cefepime neurotoxicity was considered to be the main differential. A decision was made to initiate renal replacement therapy (RRT); however, the initial placement of the dialysis catheter was compromised due to continuous oozing from the catheter site, requiring blood transfusion. A discussion regarding removal of the catheter and placing it in an alternate site ensued with the patient’s husband; however, he requested no more invasive procedures and opted for conservative management.

At this point, aggressive intravenous fluid resuscitation at 150 cc/h of normal saline was initiated with daily intravenous diuresis with furosemide. With the limitation of reduced glomerular filtration rate, we expected a prolonged course of treatment. Over the next three days, this patient’s toxic/metabolic encephalopathy resolved and she returned to baseline neurological function. Furthermore, her AKI progressively improved. This approach proved effective but initially challenging as the patient had already received a total of 6 g of cefepime with a chronically reduced glomerular filtration rate (GFR).

She was then transferred to the inpatient hospitalist service for continued management of pneumonia. Both the patient and spouse were provided with a detailed explanation of the events leading to her medical complication. Additional emphasis was placed on the importance of listing medication intolerance and she chronically compromised renal function in future medical encounters. Furthermore, the patient was advised to not receive treatment with cephalosporins, more specifically cefepime. She was then discharged home after an additional five days of hospitalization.

## Discussion

Cefepime is a parenteral fourth-generation cephalosporin that is often used clinically to treat community-acquired and hospital-acquired pneumonia due to its broad anti-microbial effect on both Gram-positive organisms including methicillin-sensitive *Staphylococcus aureus* and Gram-negative organisms such as *Pseudomonas aeruginosa* [2]. It exerts its effect by binding to penicillin-binding proteins and ultimately disrupting cell wall synthesis [3]. It also is stalwart against many of the plasmid and chromosome-mediated beta-lactamases and has increased activity against Enterobacteriaceae that are generally resistant to third-generation cephalosporins [2,4]. Various randomized control trials in patients with moderate to severe community-acquired pneumonia who had a poor response to monotherapies with penicillin or cephalosporins displayed good clinical response to cefepime. The preferred dosing is generally 1-2 g intravenously over thirty minutes twice a day with maximum plasma concentrations being two to three times higher with intravenous use than when used intramuscularly [2,3]. With respect to the pharmacokinetics of the drug, cefepime is renally eliminated with a half-life of 2-2.5 h and volume distribution of 13-22 L in healthy adults [3,4]. The most common adverse effects include headache, nausea, diarrhea, rash, fever, erythema, and colitis [2,3].

In critically ill patients who have renal failure, plasma trough concentrations are highly variable increasing the risk of toxicity [5]. In rare circumstances, cefepime has been linked to neurological side effects due to its ability to readily cross the blood-brain barrier. Adverse reactions can be further potentiated in patients with renal insufficiency due to decreased drug clearance [5,6]. This has been postulated to occur through a concentration-dependent effect on gamma-aminobutyric acid type A (GABA-A) receptor modulation [4-6]. One center reported these neurological adverse effects to occur in only 0.2% of patients receiving cefepime [6]. The most common observed clinical symptoms were diminished level of consciousness (80%), disorientation/agitation (47%), and myoclonus (40%), with a small subset of patients experiencing seizures and non-convulsive status epilepticus [6,7]. The patient presented in this case had a mixed and progressive clinical presentation of neurotoxicity as she experienced diminished level of consciousness that progressed to a comatose state and myoclonic jerks that increased in frequency until intervention.

A retrospective cohort study published in the European Society of Clinical Microbiology and Infectious Diseases found cefepime-induced neurotoxicity to occur in 100% of patients who had cefepime trough concentrations above 38.1 mg/L with the highest proportion of cases occurring in patients who had an effective glomerular filtration rate less than 30 mL/min [5]. We did not obtain an actual cefepime trough level in our case. However, having the knowledge that our patient received 6 grams of cefepime over 36 h with a baseline GFR of 24mL/min, it was fair to assume that her symptoms were caused by cefepime 

The diagnostic criteria for cefepime-induced neurotoxicity are as follows: neurological symptoms occurring several days after cefepime initiation, electroencephalogram (EEG) findings consistent with a generalized periodic discharge with a triphasic appearance, symptoms and abnormal EEG findings that resolve several days of discontinuing cefepime, no other underlying cause of metabolic or toxic encephalopathy that is likely to be the cause of the patient's symptoms, and lastly, abnormally high concentrations of cefepime [7]. Frequent monitoring of EEG, the patient's renal function, and plasma cefepime levels are recommended in patients at high risk for cefepime-induced neurotoxicity [7]. The only definitive treatment is the discontinuation of cefepime, which results in a 90% recovery rate in one to three days as seen in the case above. In emergency situations, hemodialysis can also be initiated to rapidly remove cefepime from the cerebrospinal fluid (CSF) and plasma, as a single 3-h session can remove up to 70% of a given dose [7]. Interestingly, in patients who had received anti-convulsive therapy, there was no improvement in mental status, however, there was evidence of a temporary slowing in the generalized periodic discharge on the EEG [7]. This further suggests that there is no beneficial role in adding anticonvulsive therapy [7].

The approach to this case was rather unique due to the occurrence of unforeseen events. The initial plan after establishing the diagnosis of cefepime-induced neurotoxicity was to initiate hemodialysis. However, after the placement of the hemodialysis (HD) catheter, the patient had continuous large volume blood loss from the catheter site requiring blood transfusion. It was at that point that the patient’s spouse requested no additional invasive procedures. Our treatment focus then switched to fluid administration with daily diuresis with furosemide. Over the course of 72 h, the patient progressively improved with stepwise resolution of her symptoms including resolution of mentation, respiratory function, and neural control of micturition.

## Conclusions

Cefepime-induced neurotoxicity is an under-recognized event that can present with a myriad of neurological findings ranging from myoclonus to seizures to comatose state. It is crucial for clinicians to be able to identify patients on cefepime therapy who subsequently develop new-onset neurological symptoms especially if they have underlying chronic kidney disease as early recognition can prevent unnecessary testing and therapy, and management can be as simple as prompt discontinuation of cefepime, which has a favorable outcome in this process.
